# TeleCOVID Hessen: Implikationen für die Erschließung neuer Indikationsgebiete

**DOI:** 10.1007/s00063-024-01107-1

**Published:** 2024-01-24

**Authors:** Anne Grahlmann, Jenny Brandt, Daniela Salzmann, Felix Hoffmann

**Affiliations:** 1 APOLLON Hochschule der Gesundheitswirtschaft GmbH, Universitätsallee 18, 28359 Bremen, Deutschland; 2 D&ICT, Innovation Management, Universitätsspital Basel, Basel, Schweiz

**Keywords:** SARS-CoV-2, COVID-19, Telemedizin, Intensivmedizin, Pandemie, SARS-CoV-2, COVID 19, Telemedicine, Intensive care, Pandemic

## Abstract

**Hintergrund:**

Während der Severe Acute Respiratory Syndrome Coronavirus 2-Pandemie (SARS-CoV-2-Pandemie) wurde in Hessen die Anwendung TeleCOVID entwickelt, um die Intensivstationen telemedizinisch zu vernetzen. Nach der erfolgreichen Implementierung sollte die Anwendung auch auf weitere Indikationsgebiete in der Intensivmedizin ausgeweitet werden.

**Ziel der Arbeit (Fragestellung):**

Im Rahmen dieser Studie wurde evaluiert, auf welche weiteren Indikationen der Einsatz der Anwendung erweitert werden kann und welche technischen Anforderungen damit einhergehen.

**Material und Methoden:**

Zur Beantwortung der Fragestellung wurden leitfadengestützte Experteninterviews mit klinisch tätigen Ärztinnen und Ärzten (*n* = 8) geführt, die mittels einer qualitativen Inhaltsanalyse ausgewertet wurden.

**Ergebnisse:**

Im Rahmen der Interviews konnte gezeigt werden, dass die Ausweitung von TeleCOVID auf weitere intensivmedizinische Indikationsgebiete sinnvoll ist. Es wurden zahlreiche technische Anforderungen formuliert, die bei der Weiterentwicklung der Anwendung konkret berücksichtigt werden sollten.

**Diskussion:**

Die telemedizinische Vernetzung von Intensivstationen kann für die beteiligten Akteure einen Mehrwert generieren. Wichtig ist allerdings, dass die Daten bestmöglich standardisiert und strukturiert erhoben werden. Der Kommunikationsprozess sollte umfassend automatisiert werden, um den Aufwand für die beteiligten Akteure möglichst gering zu halten.

## Abstract (en)

**Background:**

During the SARS-CoV‑2 pandemic, the TeleCOVID application was developed in Hessen to connect the intensive care units via telemedicine. After successful implementation, the application should be extended to other indication areas in intensive care medicine.

**Objectives:**

The purpose of this study was to evaluate other indications for which the application can be used and which technical requirements are associated with this.

**Materials and methods:**

To answer these questions, guideline-based expert interviews were conducted, which were evaluated using a qualitative content analysis.

**Results:**

The survey showed that TeleCOVID can be extended to other indication areas in intensive care. Numerous technical requirements were formulated that should be specifically considered when the application is further developed.

**Conclusions:**

The telemedical networking of intensive care units generates added value for the actors involved. However, it is important that the data is collected in the best possible standardized and structured way. The communication process should be automated wherever possible to minimise the workload for the participating persons.

## Einleitung

Um die hessischen Intensivstationen bei der Behandlung von Patienten mit COVID-19 zu unterstützen, wurde die Anwendung „TeleCOVID Hessen“ (Awesome Technologies Innovationslabor GmbH, Würzburg, Deutschland) entwickelt. Diese App wurde per behördlicher Anordnung vom 16.02.2021 in allen Krankenhäusern in Hessen zur Pflicht gemacht.

Im Rahmen dieser Studie wurde untersucht, welche zusätzlichen Anwendungsbereiche durch die App erschlossen werden können und welche Anforderungen dies an ihre Weiterentwicklung stellt. Das Ziel ist es, die Benutzerfreundlichkeit zu verbessern und die speziellen Anforderungen neuer Einsatzgebiete in die App zu integrieren.

## Hintergrund und Fragestellung

Um die Intensivstationen Hessens bei der Versorgung von Patienten mit der „coronavirus disease 2019“ (COVID-19) zu unterstützen, wurde die App „TeleCOVID Hessen“ (TeleCOVID) entwickelt und per Allgemeinverfügung vom 16.02.2021 verpflichtend in allen Krankenhäusern Hessens eingeführt [10]. Dies ermöglichte den Intensivstationen einen unkomplizierten und datenschutzrechtlich abgesicherten Austausch mit anderen Intensivstationen. Die App wurde aufgrund der großen Diversität der bestehenden Krankenhausinformationssysteme ohne eine KIS-Schnittstelle entwickelt. Stattdessen können Fotos (eingeordnet in ein Kategoriensystem, z. B. Blutgasanalyse, Beatmung oder Perfusoren) und Nachrichten erstellt und verschickt werden. Außerdem kann ein Austausch über Videotelefonie erfolgen. So können Telekonsile angefragt werden, um Zweitmeinungen zur Behandlung zu erhalten oder Verlegungsanfragen zu stellen [8]. Da dieser Informationsaustausch nach Patienten gegliedert dargestellt wird, ist es auch anderen Ärzten (bei Schichtwechsel oder Krankheitsvertretung) möglich, die bisherige Kommunikation nachzuvollziehen.

Es ist grundsätzlich davon auszugehen, dass die Telemedizin aufgrund des zunehmenden Fachkräftemangels zukünftig einen wachsenden Beitrag in der Gesundheitsversorgung einnehmen wird [4]. Da TeleCOVID in erster Linie für die Versorgung von SARS-CoV-2-Patienten entwickelt wurde, stellte sich die Frage, ob durch eine Weiterentwicklung der App weitere Einsatzfelder wie beispielsweise die Sepsisbehandlung erschlossen werden könnten [2]. Eine erste Evaluation der App stellte die Funktionalität der App bereits vor und zeigte auf, welche technischen Verbesserungen erforderlich sind, um die Nutzerfreundlichkeit zu erhöhen [1].

Im Rahmen der hier ergänzend durchgeführten Studie wurde untersucht, welche weiteren medizinischen Einsatzfelder durch die App erschlossen werden können und welche Anforderungen dies an die Weiterentwicklung der App mit sich bringt, um die Anwenderfreundlichkeit zu verbessern und spezielle Anforderungen neuer Indikationsfelder abzubilden.

## Studiendesign und Untersuchungsmethoden

Zur Beantwortung der Fragestellung wurden semistrukturierte Experteninterviews geführt. Bei der Entwicklung des Interviewleitfadens wurde an die Erkenntnisse von Brandt et al. [1] angeknüpft. Die Fragen wurden so formuliert, dass potenzielle neue Indikationsgebiete, Fragen bezüglich der Nutzerfreundlichkeit und weitere Anregungen teilstandardisiert erhoben wurden (vgl. Tab. 1).

**Tab. 1:** Interviewleitfaden für die Befragung

Themencluster	Potenzielle Indikationsgebiete	Notwendige Veränderungen	Weitere Anregungen
Einzelfragen	Wohin verlegen Sie regelmäßig intensivpflichtige Patienten? Bei welchen Indikationen mussten Sie vor der Coronapandemie Patienten auf eine andere Intensivstation verlegen? Fallen Ihnen außer COVID-19 noch andere Indikationen ein, bei denen Sie schon einmal eine konsiliarische Beratung a) gebraucht haben (Regelversorger), b) erbracht haben (Maximalversorger) oder bei denen eine konsiliarische Betreuung von Vorteil sein könnte? Würden Sie die TeleCOVID-App gerne noch für andere Zwecke nutzen, außer für Konsile oder Verlegungen? (z. B. Televisiten)	Wie könnte die Erreichbarkeit der konsilgebenden Ärzte verbessert werden? Haben Sie die TeleCOVID-Web-Anwendung schon einmal genutzt? Welche Vorteile hat diese? Wenn Sie sich weitere Geräte wünschen könnten, auf denen die App läuft, welche wären das? Würden Sie die App auch auf dem privaten Smartphone installieren? Wie wichtig wäre es Ihnen, dass die App eine Anbindung an das Krankenhausinformationssystem hat? Bitte begründen Sie Ihre Aussage. Welche Stammdaten des Patienten halten Sie im Notfall für absolut notwendig? Wie könnte die Anwendung bei Notfall-Verlegungen vereinfacht werden? Welche Anamnese-Informationen halten Sie bei Standard-Verlegungen grundsätzlich für notwendig? Welche Ergebnisse der klinischen Untersuchung sollten Ihrer Meinung nach übergeben werden? Welche Ergebnisse der apparativen Diagnostik interessieren Sie? In welcher Qualität brauchen Sie CT oder Röntgenbilder? Wäre es vorteilhaft, wenn die Originalbilder übertragen werden? Die App führt als mögliche Abteilungen „Intensivmedizin“, „Pharmazeutisches Konsil“ und „COVID-19“ auf. Würden Sie diese Liste erweitern oder untergliedern? Welche zusätzlichen Informationen werden für die Verlegung in eine Früh-Reha oder ein Weaning-Zentrum benötigt?	Fällt Ihnen eine Funktion ein, die Sie bei der App vermissen? Wie denken Sie darüber, dass auf den Tablets ausschließlich die TeleCOVID Hessen App läuft? Haben Sie noch Wünsche oder Anregungen für die App, damit sie in Zukunft eine Bereicherung für Ihren Arbeitsalltag darstellen kann?

Die Rekrutierung erfolgte nach einem theoretischen Sample, um möglichst spezifische Anforderungen abzubilden. Daher wurde aus jedem der sechs hessischen Versorgungsgebiete mindestens eine Person rekrutiert, die die App regelmäßig angewendet hat. Zudem wurde darauf geachtet, dass die interviewten Personen aus Krankenhäusern der Basis-, der erweiterten wie auch der umfassenden Notfallversorgung stammen. Da nur etwa die Hälfte der hessischen Krankenhäuser die App überhaupt angewendet hat und diese in etwa drei Viertel der Fälle nur sporadisch genutzt wurde [1], bewegt sich die Anzahl der Krankenhäuser, in denen die App regelmäßig genutzt wurde, im niedrigen zweistelligen Bereich. Mit insgesamt acht repräsentativ ausgewählten Befragten dürfte die Datenbasis belastbar sein.

Alle Interviews wurden von derselben Person durchgeführt und wortwörtlich transkribiert, sodass immer von demselben Interview- und Transkriptionsstil ausgegangen werden kann. Im Anschluss an die Transkription erfolgte die Auswertung mittels qualitativer Inhaltsanalyse nach Mayring [7].

## Beschreibung der Studienteilnehmer

Insgesamt nahmen 8 Experten an der Erhebung teil. Davon waren 4 männlich. Die medizinischen Fachrichtungen umfassten Anästhesiologie (6), Intensivmedizin (7) und Neurologie (1), eine Mehrfachnennung war hier möglich. Die Anzahl der Nennungen steht hier und bei den nachfolgenden Aufzählungen jeweils in Klammern.

Zwei Experten kamen aus Krankenhäusern der Basisversorgung und 4 Experten aus Krankenhäusern der erweiterten oder auch der umfassenden Notfallversorgung. Außerdem konnte je eine Person aus einer Klinik für neurologische Frührehabilitation und aus einem Weaning-Zentrum für die Studie gewonnen werden. Alle Befragten waren Ärzte, die bereits mit TeleCOVID gearbeitet hatten. Sieben der 8 Interviews wurden telefonisch geführt. Lediglich ein Interview wurde auf Wunsch als Zoom-Videokonferenz geführt.

## Ergebnisse

Nachfolgend werden die Ergebnisse der Interviews zusammengefasst.

### Frage 1: In welchen Situationen kann die App TeleCOVID neben der COVID-19-Behandlung sinnvoll eingesetzt werden?

Für Verlegungen hin zu Weaning- oder Rehakliniken ist bereits ein Button auf der Startseite der TeleCOVID Hessen App angelegt. Alle Befragten der Krankenhäuser und des Weaning-Zentrums gaben an, dass sie Patienten von der Intensivstation zur neurologischen Frührehabilitation verlegen. Fünf von 6 Krankenhäuser gaben auch an, dass sie Patienten in Weaning-Zentren verlegen würden.

Die befragten Experten der neurologischen Rehaklinik und des Weaning-Zentrums gaben an, dass sie ihre Patienten teilweise wieder in die Ursprungsklinik zurückverlegen müssten. In diesem Fall würden beide Partner von einer Abstimmung über die App profitieren, da bei einer notwendigen Rückverlegung in die Ursprungsklinik der Patient bereits in der App angelegt wäre.

Ein Krankenhaus und das Weaning-Zentrum gaben an, Patienten in die Geriatrie zu verlegen. Zudem verlegt das Weaning-Zentrum auch in andere Rehakliniken, beispielsweise zur Behandlung von Lungenkrankheiten.

Eine Verlegung in heimatnahe Krankenhäuser sei eher selten, gaben 2 Befragte aus Krankenhäusern der umfassenden Notfallversorgung an.

Die Krankenhäuser der Basis- und der erweiterten Notfallversorgung verlegen ihre Patienten regelmäßig von der Intensivstation in Häuser mit anderen Fachrichtungen oder in Häuser einer höheren Versorgungsstufe. Die Fachrichtungen, in die sie verlegen, sind dabei ganz unterschiedlich und hängen in erster Linie davon ab, welche Fachrichtungen oder Geräte den Kliniken selbst fehlen.

Für die Fachrichtungen oder Abteilungen Neurologie/Neurochirurgie (4), Thoraxchirurgie/Herzchirurgie (3), Viszeralchirurgie (2), ARDS-Zentrum (1), operative Intensivstation (1), Onkologie (1), Urologie (1), Gefäßchirurgie (1), HNO (1), nephrologisches Zentrum (1), Kardiologie (allgemein) (1), Herzkatheterlabor (1) besteht nach Einschätzung der Befragten Bedarf für zukünftige Verlegungen. Verlegungen in die Viszeralchirurgie wurden nur von der Rehaklinik und dem Weaning-Zentrum genannt, da alle anderen Basisnotfallversorger in Hessen gemäß dem deutschen Krankenhausverzeichnis eine Viszeralchirurgie haben [3].

Drei Experten gaben an, in der Vergangenheit neurologische Konsile angefordert zu haben. Zwei Experten ließen CT-Bilder befunden. Drei Experten forderten Konsile zu Beatmungsfragestellungen an. Außerdem wurden die Gastroenterologie (1), Handchirurgie (1), HNO (1) und Infektiologie (1) als Abteilungen genannt, an die in der Vergangenheit eine Konsilanfrage gestellt wurde. Ein Experte gab zudem an, eine telefonisch-konsiliarische Beatmungsberatung durchgeführt zu haben, wenn eine Übernahme vereinbart wurde, aber aus Kapazitätsgründen erst zu einem späteren Zeitpunkt möglich war.

Diese Konsile könnten ebenfalls via TeleCOVID durchgeführt werden, jedoch gaben 4 Experten an, dass Konsile über die App nur denkbar seien, wenn die Finanzierung der Konsile geklärt sei; aktuell würden die Konsile kostenfrei durchgeführt. Ein Experte gab außerdem zu bedenken, dass eine schnelle Rückmeldung der konsiliarisch hinzugezogenen Ärzte sichergestellt sein müsse. Zwei Experten hingegen gaben an, dass Konsile ohnehin nur selten gebraucht würden.

### Frage 2: Welche Modifikationen müssen an der App vorgenommen werden, um sie sinnvoll in einem erweiterten Kontext verwenden zu können?

Alle Experten gaben an, dass die übertragenen Bilder in Befundungsqualität übertragen werden sollten und eine Übertragung in das PACS des Empfängers möglich sein sollte.

Eines der größten Probleme im Gesamtprozess sei die teilweise schlechte Erreichbarkeit der anderen Häuser über das Tablet gewesen. Folgende Vorschläge für die Verbesserung des Prozesses wurden gemacht: Pop-up-Benachrichtigung am PC (3), automatisierter Anruf auf dem Dienst- oder Funktionstelefon bei Eingang einer Konsilanfrage (2) und eine automatisch generierte SMS (2). Jeweils einmal wurden folgende Vorschläge genannt: Terminabsprache über anderes Medium (z. B. WhatsApp), Benachrichtigung im KIS mit direktem Link zur Konsilanfrage, automatisierte E‑Mail an zuständigen Arzt, Benachrichtigung aller Intensivmitarbeiter, Benachrichtigung auf Smartwatch.

Ein Experte gab an, dass er es weiterhin für sinnvoll halten würde, die angefragte Klinik telefonisch über die Konsilanfrage zu informieren. Vier andere Experten gaben allerdings an, dass sie die Patienten auch gleich am Telefon übergeben und die App gar nicht mehr nutzen würden, wenn sie ohnehin anrufen müssten.

Fünf Befragte würden es bevorzugen, die Konsilanfrage auf einem Smartphone zu empfangen und zu bearbeiten. Davon wären 3 Experten sogar damit einverstanden, dass die App auf ihrem privaten Smartphone läuft, wenn dies datenschutzrechtlich möglich sei. Drei Teilnehmer würden es ablehnen. Lediglich ein Experte empfand die Display-Größe eines Smartphones als zu gering.

Die von den Experten als erforderlich genannten Stamm- oder Patientendaten stimmten mit den von TeleCOVID abgefragten Daten überein, sodass hier kein Anpassungsbedarf besteht. Eine Telefonnummer für Rückfragen könnte als zusätzlicher Parameter abgefragt werden. Weitere Pflichtfelder wurden generell abgelehnt.

Folgende Informationen sollten nach Ansicht der Befragten bei allen Intensivpatienten abgefragt werden: Infektionsstatus (6), Beatmungssituation (6), Kreislaufsituation (4), Medikamente (4), Vorerkrankungen (3), verlegungsbegründende Diagnose (2), Allergien (2), Labor (2), Ausscheidungssituation (2), Liegedauer (1), aktuelle und zuvor bestehende Vigilanz (1), Erhebungsmethode der Kreislaufsituation (1), letzter Routineabstrich multiresistenter Erreger (1), Epikrise (1), Anzahl der Perfusoren (1), Horovitz-Quotient (1), relevante Bildgebung (1), aktuelle BGA (1), Hauptproblem und Begleitprobleme (1).

Die Experten wurden ebenfalls nach ihrer Einschätzung hinsichtlich einer Standardisierung der Anamnese gefragt. Ein Experte gab an, dass er eine Standardisierung nach Organsystemen für möglich halte. Ein anderer hingegen gab an, dass eine generelle Standardisierung nicht möglich sei. Wieder eine weitere Person sagte, dass sie zumindest in Bezug auf die apparative Diagnostik keine Standardbefunde nennen könne.

Um die App für Konsile oder Verlegungen in andere Fachgebiete zu nutzen, müsse auch die Benutzeroberfläche angepasst werden. Daher wurden die Experten gefragt, wie sie die Startseite, auf der bis jetzt nur die drei Optionen „Intensivmedizin“, „pharmakologisches Konsil“ und „COVID-19“ aufgeführt sind, verändern würden. Ein Experte gab an, dass er mit dem Button „Konsil/Zweitmeinung“ unzufrieden sei und ihn um einen weiteren Button „Verlegungen“ erweitern würde, da eine Verlegung manchmal unumgänglich sei.

Sieben Experten würden es befürworten, wenn zusätzliche Abteilungen adressiert werden könnten. Nachfolgende Abteilungen wurden genannt: Infektiologie (3), operative Intensivmedizin (2), allgemeininternistische Intensivmedizin (2), Kardiologie (1), kardiologische Intensivmedizin (1), pulmologische Intensivmedizin (1), Neurologie/Neurochirurgie (1), neurochirurgische Intensivstation (1), pädiatrische Intensivmedizin (1).

Folgende Informationen, die speziell für Verlegungen zur neurologischen Frührehabilitation oder zum Weaning relevant seien, wurden genannt: Bartel-Index (7) bzw. Frührehabilitations-Barthel-Index (1), Aufnahmeantrag (4), Pflegeüberleitungsdokumentation (4), Prognose (2), neurologischer Status (1), Antibiotikahistorie (1), detaillierte Wundinformationen (1), Ernährungssituation (1).

Als weitere Nutzergruppen neben dem ärztlichen Personal benötigen das Pflegepersonal, das Belegungsmanagement sowie der Sozialdienst einen Zugang zu der App, um Verlegungsdokumente einfügen oder ausfüllen zu können.

### Frage 3: Gibt es von ärztlicher Seite noch Wünsche oder Anregungen bezüglich der App TeleCOVID, damit diese einen echten Mehrwert bietet?

Vier Experten haben angeregt, eine smarte Benutzerführung in die App zu integrieren, die den Benutzer durch den Prozess leitet. Dabei sollten Notfälle von nicht zeitkritischen Anfragen abgegrenzt und je nach Indikation gegebenenfalls unterschiedliche Workflows implementiert werden und die Workflows eine gewisse Flexibilität zulassen, um den individuellen Anforderungen gerecht zu werden.

Die Mehrzahl der interviewten Ärzte erhofft sich, dass durch eine KIS-Anbindung Daten und Befunde schneller in die App übernommen werden können. Einige der Befragten äußerten den Wunsch, dass die Konsilerstellung teilautomatisiert erfolgen könnte und der Bericht durch wenige Klicks erstellt werden kann, wobei benötigte Daten und Befunde aus dem KIS eingefügt werden, wenn möglich („Wenn ich jetzt allerdings, so wie ich das mit dem Arztbrief mache, wenn ich da Laborwerte im Arztbrief haben will, die markiere ich im Laborbefund, im Kumulativbefund, und dann sind die im Arztbrief drin. Das sind zwei Klicks.“).

Einen weiteren großen Mehrwert würde die App nach Einschätzung aller Experten der Krankenhäuser der Basis- und erweiterten Notfallversorgung und des Weaning-Zentrums erhalten, wenn die Daten, die über eine KIS-Verbindung nicht nur als Fotos, sondern als reale Daten vorlägen, in die Anwendung „Rescuetrack“ importierbar wären.

Schließlich wäre die Entwicklung eines standardisierten Aufnahmebogens für die neurologische Frührehabilitation ein Mehrwert, da im Moment jede Klinik ihr eigenes Formular habe, das ausgefüllt werden müsse. Von der Expertin der neurologischen Frührehabilitation wird die Entwicklung eines solchen Bogens als möglich eingeschätzt. Sie gab allerdings zu bedenken, dass Besonderheiten, wie die Notwendigkeit einer Dialyse oder Isolation, berücksichtigt werden müssten, da nicht jede Klinik dies leisten könne.

## Diskussion

Im Rahmen dieser Studie konnte gezeigt werden, dass es neben COVID-19 auch weitere Indikationen für die Abstimmung von Intensivstationen untereinander gibt. Demnach kann angenommen werden, dass hessische Intensivstationen grundsätzlich auch bei anderen Indikationen von einer telemedizinischen Vernetzung profitieren würden. Am häufigsten wurden Verlegungen in die Neurologie/Neurochirurgie oder in die Herz- und Thoraxchirurgie genannt, was sich auch mit Erkenntnissen aus der Literatur deckt [9].

Auf Datenebene zeigte sich, dass es einerseits Daten gibt, die unabhängig von der Indikation immer erforderlich sind, wie beispielsweise Name, Geburtsdatum und Hauptdiagnose. Darüber hinaus gibt es Daten, die nur bei bestimmten Indikationen erforderlich sind. Bei der Erweiterung auf andere Indikationen sollte diese Flexibilität berücksichtigt werden. Denkbar wären beispielsweise je nach Indikation spezielle Fragebögen (z. B. für Weaning-Verlegungen oder für die neurologische Frührehabilitation).

Es ist von essenzieller Bedeutung, dass die übermittelten Daten bestmöglich strukturiert sind und bestenfalls strukturell in dem Format ausgegeben werden, das der jeweilige Empfänger auch für eigene Patienten kennt. In der Notfallmedizin hat sich in den letzten Jahren das AUD_2_IT-Schema als Instrument für eine Prozess- und Datenstrukturierung etabliert, in das alle relevanten Parameter strukturiert und flexibel integriert werden könnten und das hier ebenfalls sinnvoll anwendbar sein dürfte [5, 6].

Grundsätzlich würde die Anwendung von einer möglichst umfassenden Automatisierung aller Prozessschritte profitieren. So sollten beispielsweise die Stammdaten aus dem KIS exportiert werden und die Alarmierung des konsilgebenden Arztes automatisch erfolgen, beispielsweise mittels Push-Nachricht. Parallel durchgeführte Anrufe führen zu einem unnötigen Zeitverlust.

Insgesamt decken sich die Ergebnisse mit der vorhergehenden Studie von Brandt et al. [1], in der die Anwendererfahrung des Projekts „TeleCOVID Hessen“ evaluiert wurde. Dennoch sind die Aussagen dieser Studie stellenweise limitiert. Mittels der Auswahlkriterien der Teilnehmer wurde versucht, ein breites Spektrum an Anforderungen zu erheben. Allerdings kann es sein, dass aufgrund der geringen Teilnehmerzahl von 8 Personen spezifische Bedarfe einzelner Versorgungsbereiche nicht erfasst worden sind. Methodisch wurde zwar ein Interviewleitfaden verwendet, aufgrund der großen Variabilität der Interviews kamen einzelne Aspekte jedoch nur in einem Teil der Interviews zur Sprache.

Trotz dieser Limitationen konnten durch diese Studie die Anforderungen an die TeleCOVID-App umfassend erhoben und hilfreiche Informationen zur Weiterentwicklung der App erhoben werden, damit zukünftig auch andere Anwendungsfelder von dieser Entwicklung profitieren können. Eine Umsetzung dieser Empfehlungen ist nicht mehr Bestandteil dieser Studie und liegt in der Verantwortung der Herstellerfirma.

## Fazit für die Praxis

- Die telemedizinische Vernetzung von Intensivstationen könnte neben COVID-19 auch bei anderen Indikationen einen Mehrwert bieten.
- Auf der technischen Seite ist es wichtig, dass die Daten strukturiert erhoben und automatisiert übermittelt werden, um den Zeitaufwand für alle Beteiligten so gering wie möglich zu halten.
- Die Datenstrukturierung sollte anhand anerkannter Standards wie des AUD_2_IT-Schemas erfolgen.
